# Elucidating Trigonelline's Therapeutic Mechanisms for Traumatic Brain Injury Through Integrated Network Pharmacology and In Vivo Validation

**DOI:** 10.1002/cns.70803

**Published:** 2026-03-09

**Authors:** Qian Zhang, Yuefan Zhang, Zhibing Song, Zixiang Tang, Guoqiang Wang, Xiang Yang, Jincai Li, Tiejun Li

**Affiliations:** ^1^ School of Traditional Chinese Medicine Bozhou University Bozhou China; ^2^ Department of Pharmacy Punan Hospital Shanghai China; ^3^ School of Medicine Shanghai University Shanghai China; ^4^ School of Biological and Medical Engineering Donghua University Shanghai China; ^5^ Zhangjiagang TCM Hospital Affiliated to Nanjing University of Chinese Medicine Suzhou China

**Keywords:** MAPK pathway, molecular docking, network pharmacology, traumatic brain injury, trigonelline

## Abstract

**Background:**

Traumatic brain injury (TBI) triggers complex pathological cascades, including inflammation, oxidative stress, apoptosis, and gliosis, particularly during the acute phase after injury. Trigonelline has been reported to exert neuroprotective effects in experimental models; however, its molecular mechanisms in acute TBI remain insufficiently defined.

**Objective:**

This study aimed to elucidate the molecular targets and mechanisms by which trigonelline attenuates acute TBI using an integrated network pharmacology and experimental validation approach.

**Methods:**

A trigonelline–target interaction network was constructed based on network pharmacology, followed by GO/KEGG analyses to predict the biological processes and pathways involved. Molecular docking was conducted to validate the binding affinity of trigonelline with key targets. Animal experiments were carried out to confirm the mechanistic predictions.

**Results:**

Network pharmacology identified GAPDH, IL6, ALB, TNF, and IL1B as major hub genes associated with trigonelline. GO/KEGG analyses suggested that the neuroprotective effects of trigonelline against TBI primarily involved the MAPK and PI3K‐Akt pathways. In vivo assays demonstrated that trigonelline treatment significantly reduced brain water content, inflammation, and oxidative stress levels within 72 h post‐injury, while ameliorating histopathological damage, as confirmed by ELISA, HE, and LFB staining. TUNEL, NeuN, and FJB staining further revealed that trigonelline attenuated TBI‐induced neuronal apoptosis. Western blotting demonstrated that trigonelline suppressed MMP‐9 and AQP4 expression and attenuated the triggering of the MAPK signaling pathway.

**Conclusion:**

By attenuating MAPK signaling and apoptosis, trigonelline mitigates neural damage following TBI. The present findings provide experimental evidence supporting the neuroprotective effects of trigonelline in an acute TBI model.

## Graphical abstract

Trigonelline alleviates traumatic brain injury (TBI) by attenuating oxidative stress and neuroinflammation. It inhibits MAPK signaling–mediated neuronal apoptosis and reduces cerebral edema via downregulation of AQP4 and MMP‐9, thereby maintaining blood–brain barrier integrity. These results suggest that targeting the MAPK pathway underlies the neuroprotective effects of trigonelline in TBI.

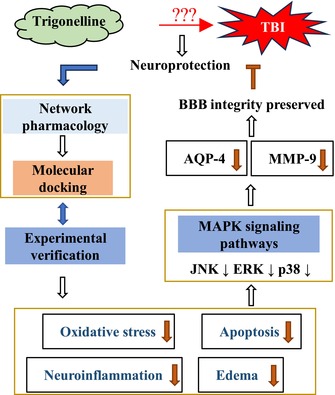

## 1 Introduction

TBI is brain damage caused by external mechanical impact or violence. It represents a leading cause of epilepsy, psychiatric disorders, and other chronic neurological conditions, and additionally serves as a principal factor contributing to global mortality and disability [1, 2]. Patients with TBI frequently experience motor, sensory, and cognitive impairments, resulting in sustained deterioration in quality of life and imposing substantial social and economic burdens [3]. The pathology of TBI involves both primary injuries, such as cerebral contusion and intracranial hematoma, and secondary injuries characterized by neuroinflammation, oxidative stress, apoptotic cell death, and disruption of the blood–brain barrier (BBB) [4, 5]. Despite continuous advances in emergency care and rehabilitation, effective therapeutic strategies for TBI remain limited, underscoring the need for novel therapeutic strategies targeting secondary injury cascades during the acute phase.

In recent years, natural agents and pharmacologically active molecules derived from traditional Chinese medicine (TCM) have received heightened attention for TBI research owing to their multitarget and multipathway regulatory properties [6]. Trigonelline (TG), a major alkaloid found in fenugreek ( *Trigonella foenum‐graecum* ), *Leonurus japonicus* (motherwort), and other medicinal and edible plants, has been reported to exhibit diverse biological activities, including anti‐apoptotic, anti‐inflammatory, antioxidant, neuroprotective, and cognition‐enhancing effects [7, 8]. TG has been reported to alleviate neuronal injury by modulating inflammatory responses, scavenging reactive oxygen species (ROS), and supporting mitochondrial function [9]. It exhibits pronounced neuroprotective properties in preclinical studies investigating neurodegenerative conditions, such as Alzheimer's and Parkinson's disease [10, 11]. Furthermore, TG mitigates lipopolysaccharide (LPS)‐induced neuroinflammatory responses by boosting endogenous antioxidant capacity, including superoxide dismutase (SOD) and glutathione, while reducing lipid peroxidation and pro‐inflammatory cytokines (TNF‐α, IL‐6) [12]. Given the pivotal roles of oxidative stress and inflammation in secondary brain injury following TBI, TG represents a promising candidate for further mechanistic investigation in acute TBI.

Network pharmacology has emerged as an innovative approach in drug discovery by integrating compound screening, target prediction, and pathway enrichment analyses to construct potential drug–target networks, which can subsequently be validated in vivo to enhance mechanistic insight and translational relevance [13, 14]. Complementarily, molecular docking enables simulation of compound–target interactions, providing information on binding modes and affinities that support target validation and mechanistic exploration [15]. Together, these approaches are particularly well suited for elucidating the complex mechanisms of action of TCM‐derived bioactive compounds.

This study employed network pharmacology to predict candidate protein targets of TG for TBI therapy, followed by functional enrichment analyses to clarify its mechanistic basis. The potential binding of trigonelline to these shortlisted targets was then modeled and scored through molecular docking. Ultimately, a mouse model of acute TBI was applied to validate the predicted mechanisms and to investigate the neuroprotective effects of TG during the early post‐injury period.

## 2 Materials and Methods

### 2.1 Network Pharmacology‐Based Analysis

#### 2.1.1 Collection and Screening of Trigonelline‐ and TBI‐Related Targets

We retrieved the TG‐associated targets from the TCMSP database and queried the PubChem database for its PubChem ID and SMILES notation. Targets prediction was performed using BATMAN‐TCM (score cutoff ≥ 20, *p* ≤ 0.05) and SwissTargetPrediction (STP). The chemical structure of TG was uploaded to STP to identify putative targets. All predicted targets were standardized to official gene names using UniProt and restricted to *Homo sapiens* . Potential TBI‐associated targets were collected from the GeneCards and OMIM databases using “traumatic brain injury” as the search term. Overlapping targets between TG and TBI were mapped using Venny 2.1.

#### 2.1.2 PPI Network Construction

A PPI analysis was conducted on the intersecting targets via the STRING platform, limited to *Homo sapiens* with a minimum interaction score of 0.4. Isolated nodes were removed to enhance network reliability. Cytoscape 3.10.3 was used for visualization. Using the MCODE and cytoNCA plugins, critical modules and hub genes were discovered, from which the top 20 were designated as core targets based on their network topology characteristics.

#### 2.1.3 GO/KEGG Analysis

The functional profiling of the core targets was explored through enrichment analysis. This was done using the Metascape database to interrogate Gene Ontology (GO) and Kyoto Encyclopedia of Genes and Genomes (KEGG) pathways. A *p* < 0.01 was set as the cutoff criterion. The top 20 enriched terms were ranked according to −log (*p*) values and visualized as bar plots (GO) and bubble plots (KEGG), with emphasis on key biological processes and signaling pathways.

#### 2.1.4 Molecular Docking Analysis

Core targets identified by CytoHubba using the Maximal Clique Centrality (MCC) algorithm were subjected to molecular docking with TG. We sourced TG's two‐dimensional structure from PubChem, refined it with Chem3D, and retrieved the crystal structures of all target proteins from the PDB. Docking simulations were conducted using Discovery Studio 2019. The docking outcomes were rendered in 3D using PyMOL, while two‐dimensional ligand–protein interaction diagrams were generated with LigPlot.

### 2.2 Materials and Experimental Animals

**FIGURE 1:**
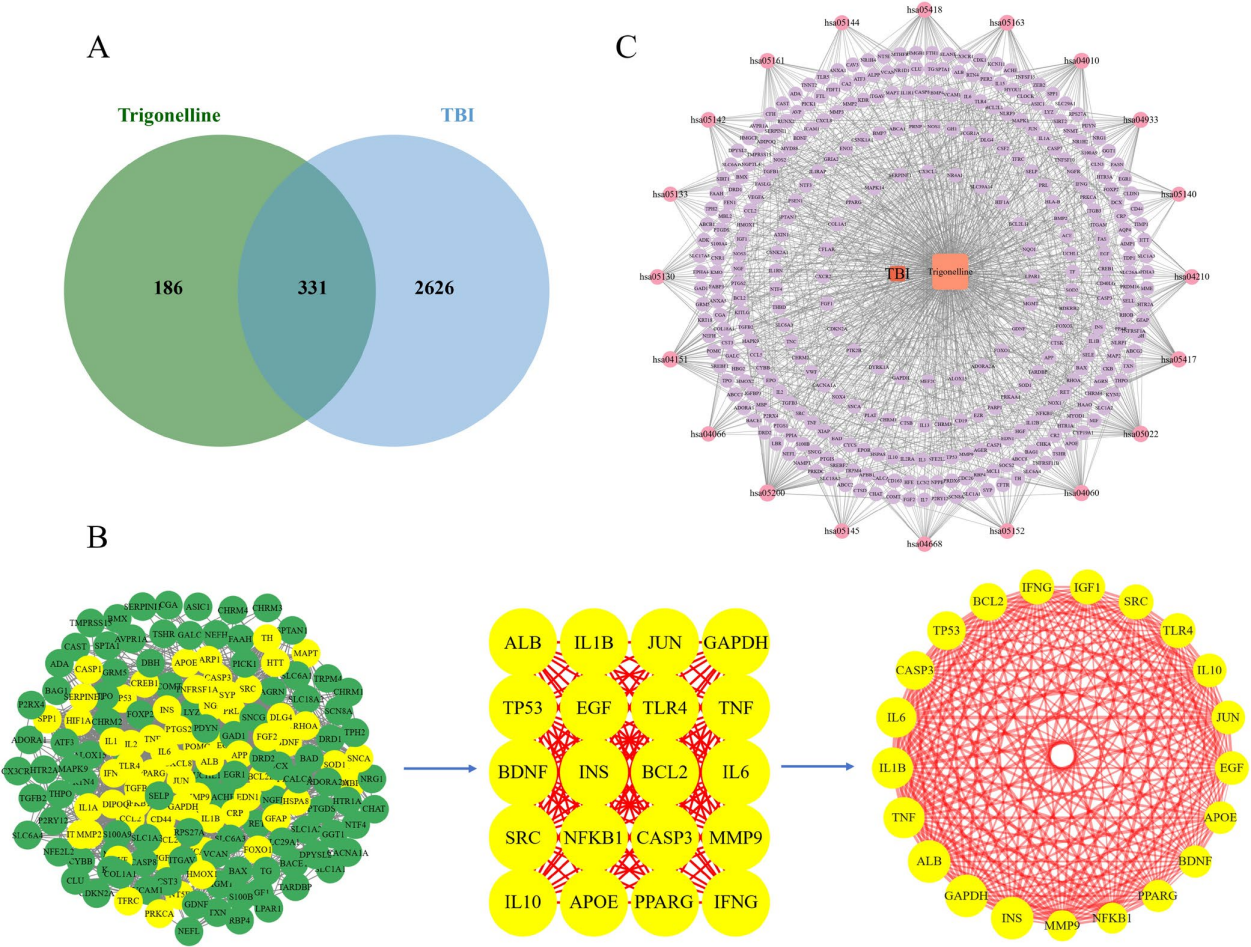
Predicted targets of TG for TBI treatment based on network pharmacology. (A) Venn diagram showing the overlapping targets between TG and TBI. (B) PPI network of the overlapping targets. (C) “TG–target–pathway–TBI” network.

Edaravone (EDA) was purchased from China National Pharmaceutical Group Guorui Pharmaceutical Co. Ltd. (approval number: H20080592). TG hydrochloride (C7H7NO2·HCl, CAS: 6138‐41‐6) was obtained from Tokyo Chemical Industry Co. Ltd., and its chemical structure is displayed in Figure 1B.

We acquired male BALB/c mice, aged 8–9 weeks and weighing 18–20 g, from Shanghai Sipul‐Bikai Laboratory Animal Co. Ltd. (license: SCXK (Hu) 2016–0003). Following a 7‐day acclimation period in a controlled environment (22°C–25°C, 70% humidity, natural light/dark cycle) with free access to food and water, the experiments commenced.

### 2.3 TBI Model

All animal procedures were conducted in accordance with approval from the Institutional Animal Care and Use Committee (ECSHU‐2024‐005). Mice were anesthetized with pentobarbital sodium and positioned prone. To induce TBI, a 20 g weight was allowed to fall freely from 50 cm through a vertical PVC tube (20 mm diameter, 1.5 m length) onto the exposed dura mater (Figure 1A). Successful TBI induction was indicated by decerebrate rigidity and respiratory suppression. Postoperatively, mice were placed in a thermoregulated environment until recovery. Animals were randomly allocated to five groups: Sham, TBI, TBI + TG (35 mg/kg), TBI + TG (140 mg/kg), and TBI + EDA (2 mg/kg). All assessments and tissue collections were performed within 72 h post‐injury.

### 2.4 mNSS

The mNSS was employed for the assessment of neurological deficits at 24, 48, and 72 h post‐TBI, which evaluates motor, sensory, reflex, balance, and external stimulus responses. Scoring ranges from 0 (normal) to 18 (severe neurological impairment) [16].

### 2.5 Brain Water Content

At 72 h post‐TBI, the wet weight (WW) of brains, excluding cerebellum and brainstem, was measured immediately, followed by drying at 95°C for 24 h to obtain dry weight (DW). Brain water content was calculated as: % = (WW—DW)/WW × 100 [17].

### 2.6 Measurement of SOD, MDA, and GSH‐Px in Brain Tissue

Fresh brain tissues from six animals per group were processed in ice‐cold PBS and centrifuged. Supernatants were then collected for the determination of SOD, malondialdehyde (MDA), and glutathione peroxidase (GSH‐Px) activity using commercial kits from Elabscience, as directed by the manufacturer.

### 2.7 Enzyme‐Linked Immunosorbent Assay (ELISA)

At 72 h post‐TBI, mice were deeply anesthetized. Terminal blood collection was then conducted through the abdominal aorta. The samples were separated by centrifugation after clotting. Levels of TNF‐α, IL‐6, and IL‐1β were measured using ELISA kits (Elabscience) following the kit instructions.

### 2.8 Histopathological Examination (HE)

Fixed brain tissues (4% PFA) were dehydrated, embedded in paraffin, and sectioned. HE staining was performed to assess histopathological changes. To specifically evaluate myelination in the corpus callosum and internal capsule, Luxol fast blue (LFB) staining was performed [18]. Images were captured using a light microscope under identical settings.

### 2.9 TUNEL Staining

To evaluate neuronal apoptosis, double immunofluorescence staining with TUNEL and NeuN was conducted. Tissue sections were permeabilized with 0.1% Triton X‐100, blocked, and incubated overnight at 4°C with anti‐NeuN antibody. After incubation with fluorescent secondary antibodies, TUNEL labeling was performed using a TdT‐mediated CY3‐dUTP reaction. Following DAPI nuclear counterstaining, fluorescence was observed and recorded using a fluorescence microscope.

### 2.10 NeuN Staining

Paraffin sections were deparaffinized, subjected to antigen retrieval in EDTA buffer (pH 9.0), blocked with 3% BSA, and incubated with primary antibodies (4°C, overnight). After incubation with fluorescent secondary antibodies and DAPI counterstaining, images were obtained using a Nikon inverted fluorescence microscope.

### 2.11 Fluoro‐Jade B (FJB) Staining

After sequential immersion in ethanol gradients (100% for 2 min, 70% for 1 min) and 0.06% potassium permanganate, sections were stained with 0.0001% Fluoro‐Jade B (FJB) in 0.1% acetic acid. Fluorescent signals were captured using a Zeiss microscope (excitation 450–490 nm).

### 2.12 Immunofluorescence Detection of MMP‐9 and Aquaporin‐4 (AQP4) Expression

Sections were deparaffinized, rehydrated, antigen‐retrieved, permeabilized, and blocked prior to incubation with primary antibodies against MMP‐9 or AQP4. After incubating with fluorescent secondary antibodies and DAPI staining of nuclei, images were obtained under standardized exposure settings. Quantitative analysis was performed using ImageJ.

### 2.13 Western Blot Analysis

Proteins from peri‐lesional cortices were extracted and quantified using a BCA assay. Equal amounts of protein were separated by SDS‐PAGE and transferred to PVDF membranes. Membranes were incubated overnight at 4°C with primary antibodies against MMP‐9, AQP4, ERK, p‐ERK, p38, p‐p38, JNK, p‐JNK, and β‐Actin, followed by incubation with HRP‐conjugated secondary antibodies. Signals were detected using ImageQuant 800, and densitometries were conducted with ImageJ.

### 2.14 Statistical Analysis

Data are presented as mean ± standard error of the mean (SEM). Statistical analyses were performed using SPSS software (version 17.0). After verifying data normality and homogeneity of variance, one‐way analysis of variance (ANOVA) was employed, followed by Dunnett's post hoc test. A *p* < 0.05 was considered statistically significant. Additional data processing was performed using Prism software (version 8.0).

## 3 Results

### 3.1 Network Pharmacology Analysis

#### 3.1.1 Predicted Mechanisms of Trigonelline in the Treatment of TBI

Using the TCMSP, BATMAN‐TCM, and STP databases, a total of 517 candidate targets of TG were predicted. Concurrently, 2957 targets associated with TBI were obtained from the GeneCards and OMIM databases after duplicates were removed. Venny 2.1.0 analysis revealed 331 overlapping targets between TG and TBI (Figure 1A). These shared targets were imported into STRING to construct a PPI network (Figure 1B), containing 327 nodes and 8265 edges, with a mean node degree of 50.55. Further analysis with Cytoscape 3.7.0 and the CentiScaPe 2.2 plugin, based on topological parameters (Degree unDir > 50.550459, Betweenness unDir > 315.149847, Closeness unDir > 0.001585), identified 20 key hub targets (Figure 1B, Table 1).

**TABLE 1:** Information of 20 core targets.

No.	UniProt ID	Gene symbol	Protein name	Degree
1	P04406	GAPDH	Glyceraldehyde‐3‐phosphate dehydrogenase	224
2	P05231	IL6	Interleukin‐6	211
3	P02768	ALB	Albumin	206
4	P01375	TNF	Tumor necrosis factor (TNF‐α)	202
5	P01584	IL1B	Interleukin‐1 beta	194
6	P01308	INS	Insulin	192
7	P04637	TP53	Tumor protein p53	174
8	P42574	CASP3	Caspase‐3	165
9	P14780	MMP9	Matrix metalloproteinase‐9	160
10	P01579	IFNG	Interferon‐gamma	158
11	P12931	SRC	Proto‐oncogene tyrosine‐protein kinase Src	156
12	P10415	BCL2	B‐cell lymphoma 2 protein	152
13	P22301	IL10	Interleukin‐10	150
14	P19838	NFKB1	Nuclear factor kappa B subunit 1 (p105/p50)	147
15	Q16665	HIF1A	Hypoxia‐inducible factor 1‐alpha	146
16	P05412	JUN	Transcription factor AP‐1 (c‐Jun)	145
17	P13500	CCL2	C‐C motif chemokine ligand 2 (MCP‐1)	143
18	O00206	TLR4	Toll‐like receptor 4	143
19	P01137	TGFB1	Transforming growth factor beta‐1	142
20	P23560	BDNF	Brain‐derived neurotrophic factor	141

Furthermore, an interaction network integrating TG, the 331 overlapping targets, and 20 TBI‐related signaling pathways was constructed using Cytoscape (Figure 1C). This network provides a visual representation of the potential mechanisms by which TG may regulate biological processes associated with TBI. Different geometrical symbols were used for clarity: purple circles indicate targets, pink circles represent signaling pathways, orange squares denote trigonelline, and red squares correspond to TBI‐related pathological processes.

#### 3.1.2 GO/KEGG Analysis

**FIGURE 2:**
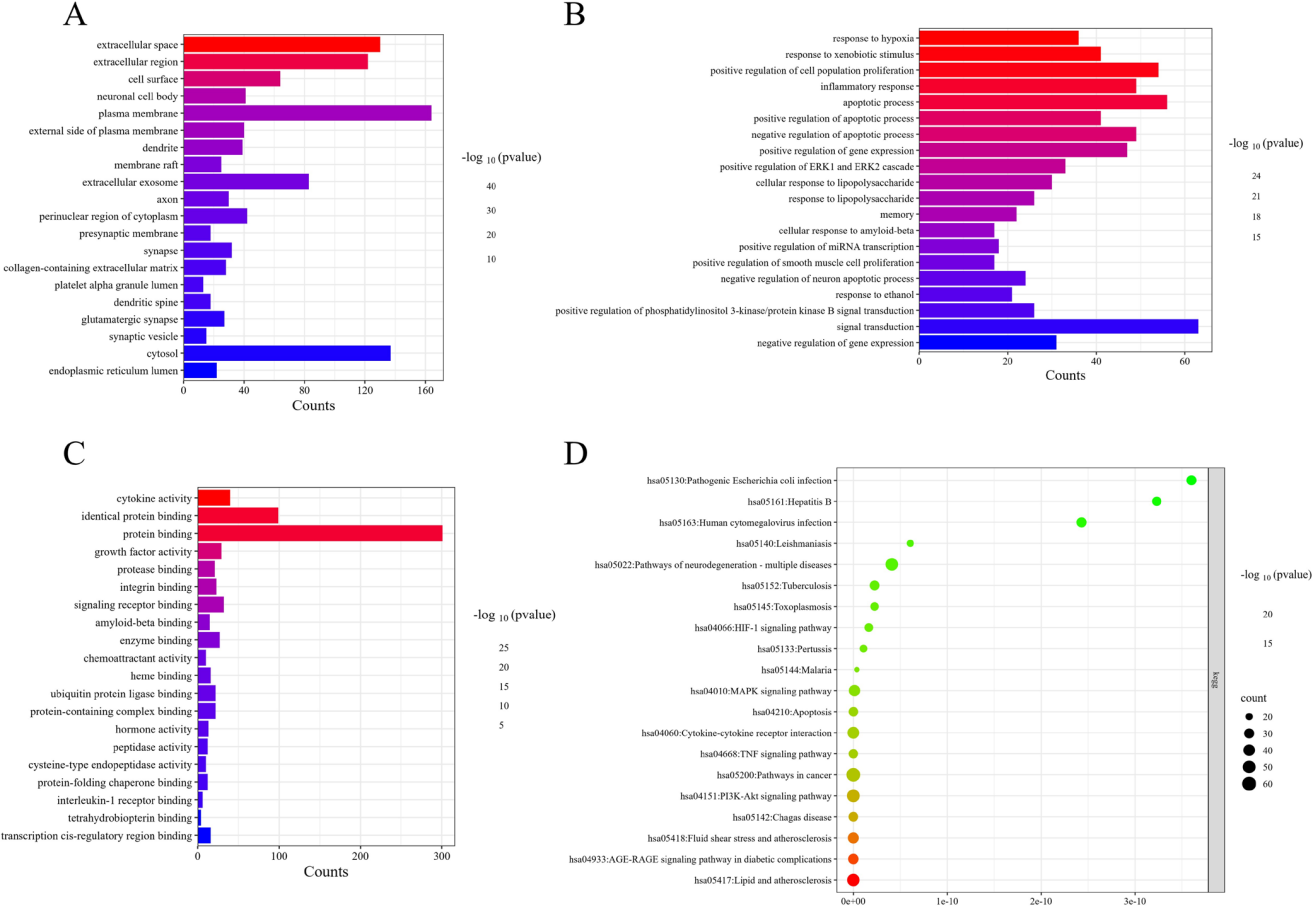
GO/KEGG analyses. (A) Top 20 CC terms. (B) Top 20 BP terms. (C) Top 20 MF terms. (D) Top 20 KEGG pathways.

The 331 intersecting genes were analyzed in Metascape to perform GO and KEGG analyses. The GO analysis highlighted the top 20 enriched terms in cellular component (CC), biological process (BP), and molecular function (MF) (Figure 2A–C). The results indicated that extracellular space and extracellular region (CC), response to hypoxia and response to external stimuli (BP), as well as cytokine activity and homodimeric protein binding (MF) were closely associated with the pharmacological actions of TG.

KEGG analysis revealed that TG primarily mediates its effects via multiple key signaling pathways, such as the MAPK, TNF, and PI3K–Akt pathways (Figure 2D, Table 2).

**TABLE 2:** Enriched KEGG pathways.

ID	Term	P Value	Count
hsa05200	Pathways in cancer	4.13E‐16	60
hsa04151	PI3K‐Akt signaling pathway	8.99E‐18	51
hsa05022	Pathways of neurodegeneration—multiple diseases	5.00E‐12	50
hsa05417	Lipid and atherosclerosis	2.87E‐25	48
hsa04060	Cytokine‐cytokine receptor interaction	2.92E‐15	43
hsa04010	MAPK signaling pathway	9.45E‐14	41
hsa05418	Fluid shear stress and atherosclerosis	5.12E‐22	37
hsa04933	AGE‐RAGE signaling pathway in diabetic complications	5.08E‐24	34
hsa05163	Human cytomegalovirus infection	3.34E‐11	32
hsa05152	Tuberculosis	2.59E‐12	30
hsa05130	Pathogenic *Escherichia coli* infection	5.50E‐11	30
hsa05142	Chagas disease	3.94E‐18	29
hsa04210	Apoptosis	1.07E‐14	29
hsa04668	TNF signaling pathway	2.47E‐15	28
hsa05161	Hepatitis B	4.68E‐11	27
hsa04066	HIF‐1 signaling pathway	1.63E‐12	24
hsa05145	Toxoplasmosis	2.44E‐12	24
hsa05133	Pertussis	9.96E‐13	21
hsa05140	Leishmaniasis	7.87E‐12	20
hsa05144	Malaria	3.14E‐13	18

#### 3.1.3 Molecular Docking Validation

**FIGURE 3:**
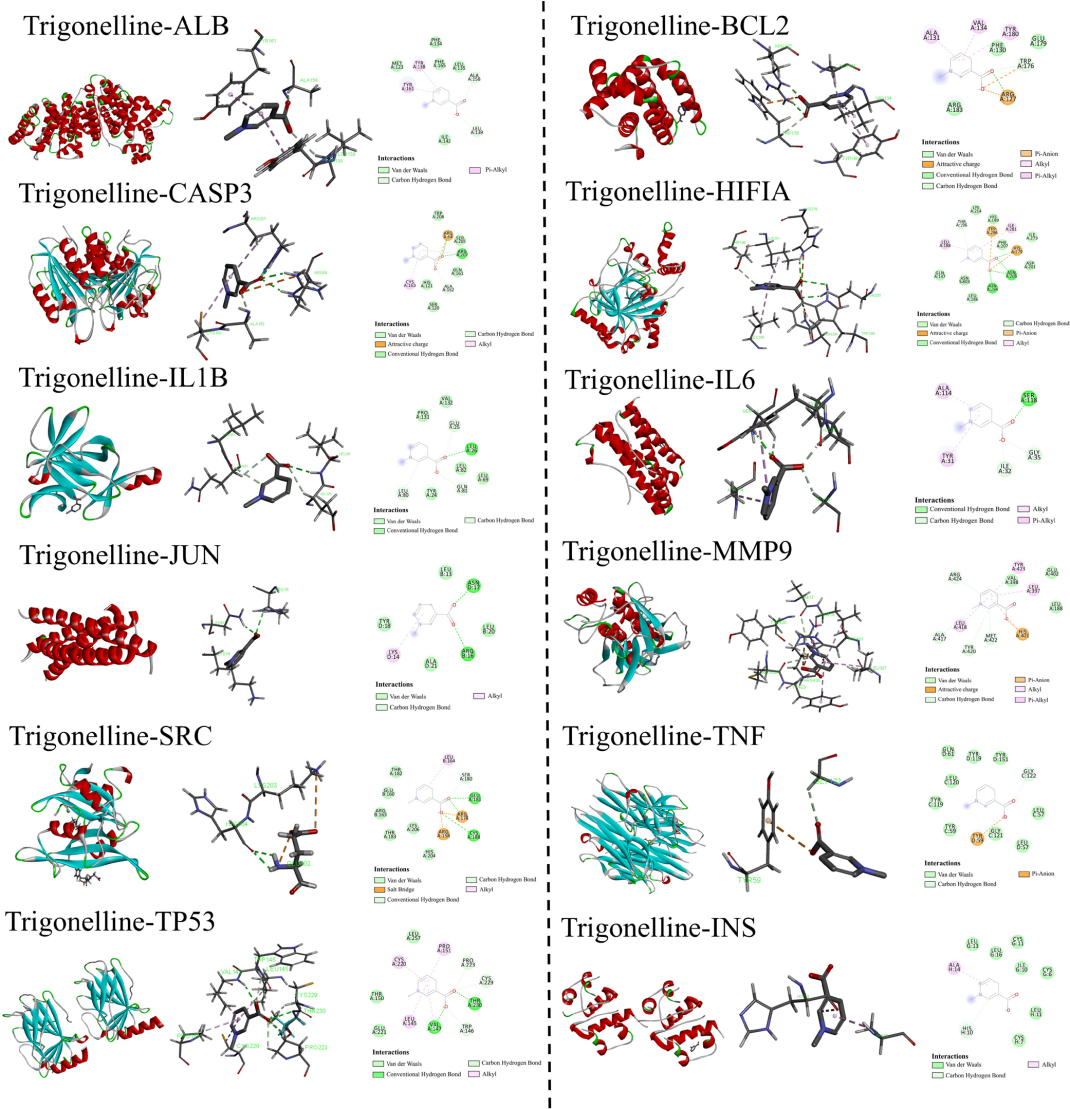
Molecular docking of TG with the top 12 key proteins (ALB, BCL2, CASP3, HIF1A, IL1B, IL6, JUN, MMP9, SRC, TNF, TP53, and INS). From left to right, the panels represent the schematic of the protein binding pocket, the distribution of amino acid residues, and the 2D representation of the TG–protein complex.

A total of 20 key targets were selected for molecular docking simulations, including ALB, BCL2, CASP3, HIF1A, IL1B, IL6, JUN, MMP9, SRC, TNF, TP53, and INS (Table 3). In docking analysis, more negative docking scores indicate stronger binding affinity [19]. As shown in Figure 3, TG formed stable hydrogen bonds with multiple targets. Specifically, TG interacted with ALB at ALA158, INS at HIS10, and TNF at GLY122. Multiple hydrogen bonds were observed with CASP3 (ARG207, ARG64), BCL2 (TRP176, ARG127), JUN (ASN17, ARG16), IL6 (SER118, ILE32, GLY35), IL1B (LEU80, GLN81, LEU26, GLU25), MMP9 (ARG424, ALA417, TYR420, MET422), SRC (SER180, GLU181, ARG178, CYS188), HIF1A (THR196, HIS279, ASN205, ASN294), and TP53 (VAL147, TRP146, THR230, CYS229, PRO223).

**TABLE 3:** Details of targets and TG for molecular docking.

Key target	PDB ID	Amino acid residue	Type of interaction	Binding energy (kcal/mol)
IL6	1ALU	SER118、ILE32、GLY35	Hydrogen bond	−4.4
		ALA114、TYR31	Alkyl	
ALB	1BJ5	ALA158	Hydrogen bond	−5.4
		TYR138、TYR161	Pi‐Alkyl	
TNF	2AZ5	GLY122	Hydrogen bond	−5.0
		TYR59	PI‐Anion	
IL1B	5R8Q	LEU80、GLN81、LEU26、GLU25	Hydrogen bond	−4.6
INS	1G7A	ALA14	Alkyl	−4.7
		HIS10	Hydrogen bond	
TP53	3ZME	CYS220、LEU145、PRO151、VAL147	Alkyl	−5.2
		VAL147、TRP146、THR230、CYS229、PRO223	Hydrogen bond	
CASP3	1NMQ	CYS163、ARG207	Alkyl	−4.9
		ARG207、ARG64	Hydrogen bond	
MMP9	1GKC	ARG424、ALA417、TYR420、MET422	Hydrogen bond	−5.9
		LEU418、HIS401、LEU397、TYR423	Alkyl	
		HIS401	PI‐Anion	
SRC	1A07	LEU164	Alkyl	−5.5
		SER180、GLU181、ARG178、CYS188	Hydrogen bond	
BCL2	6GL8	TYR180、VAL134、ALA131	Alkyl	−5.0
		TRP176、ARG127	Hydrogen bond	
		ARG127、TRP176	PI‐Anion	
HIFIA	1H2K	LEU188、ILE281	Alkyl	−5.7
		THR196、HIS279、ASN205、ASN294	Hydrogen bond	
		HIS279、TRP296	PI‐Anion	
JUN	5FV8	LYS14	Alkyl	−4.1
		ASN17、ARG16	Hydrogen bond	
CCL2	2RA4	LEU46、THR44	Conventional hydrogen bond	−4.0
		ARG24	Attractive charge	
AQP4	3GD8	HOH19、HOH260、HOH11	Water hydrogen bond	−4.6
		GLY146、GLY144、VAL141、ASN58	Conventional hydrogen bond	
JNK	3ELJ	HOH545、HOH605、HOH518、HOH507	Water hydrogen bond	−5.2
		ASN114、MET111	Hydrogen bond	
		ALA113	Van der waals	
		SER34	Halogen	
		VAL158	Pi‐sigma	
		ASP112	Amide‐pi stacked	
		VAL40、LEU168、ILE32、ALA53	Pi‐alkyl	
ERK2	1PME	MET108	Hydrogen bond	−5.2
		HOH2011、HOH2176	Water hydrogen bond	
		LEU103、VAL104	Halogen	
		THR105、LEU156	Pi‐sigma	
		LYS54、ALA52、VAL39、CYS166	Pi‐alkyl	
P38	1A9U	HIS107、MET109	Hydrogen bond	−5.2
		LYS53	Unfavorable donor‐donor	
		ASP168	Pi‐anion	
		LEU104、VAL105	Halogen	
		THR106、VAL38	Pi‐sigma	
		TYR35	Pi‐pi stacked	
		ALA51	Pi‐alkyl	
NFKB1	8TQD	ARG56	Conventional hydrogen bond	−4.3
		TYR59	Pi‐Alkyl	
GAPDH	1V8F	GLN56、GLN149	Hydrogen bond	−5.3
		HOH1966、HOH1602、HOH1752	Water hydrogen bond	
		CL1305	Attractive charge	

The results imply that TG has favorable binding potential with multiple inflammation–, apoptosis–, and barrier‐related targets such as ALB, BCL2, CASP3, HIF1A, and IL1B, providing a molecular basis for its predicted regulatory effects in TBI.

### 3.2 Trigonelline Improves Neurological Dysfunction Following TBI

**FIGURE 4:**
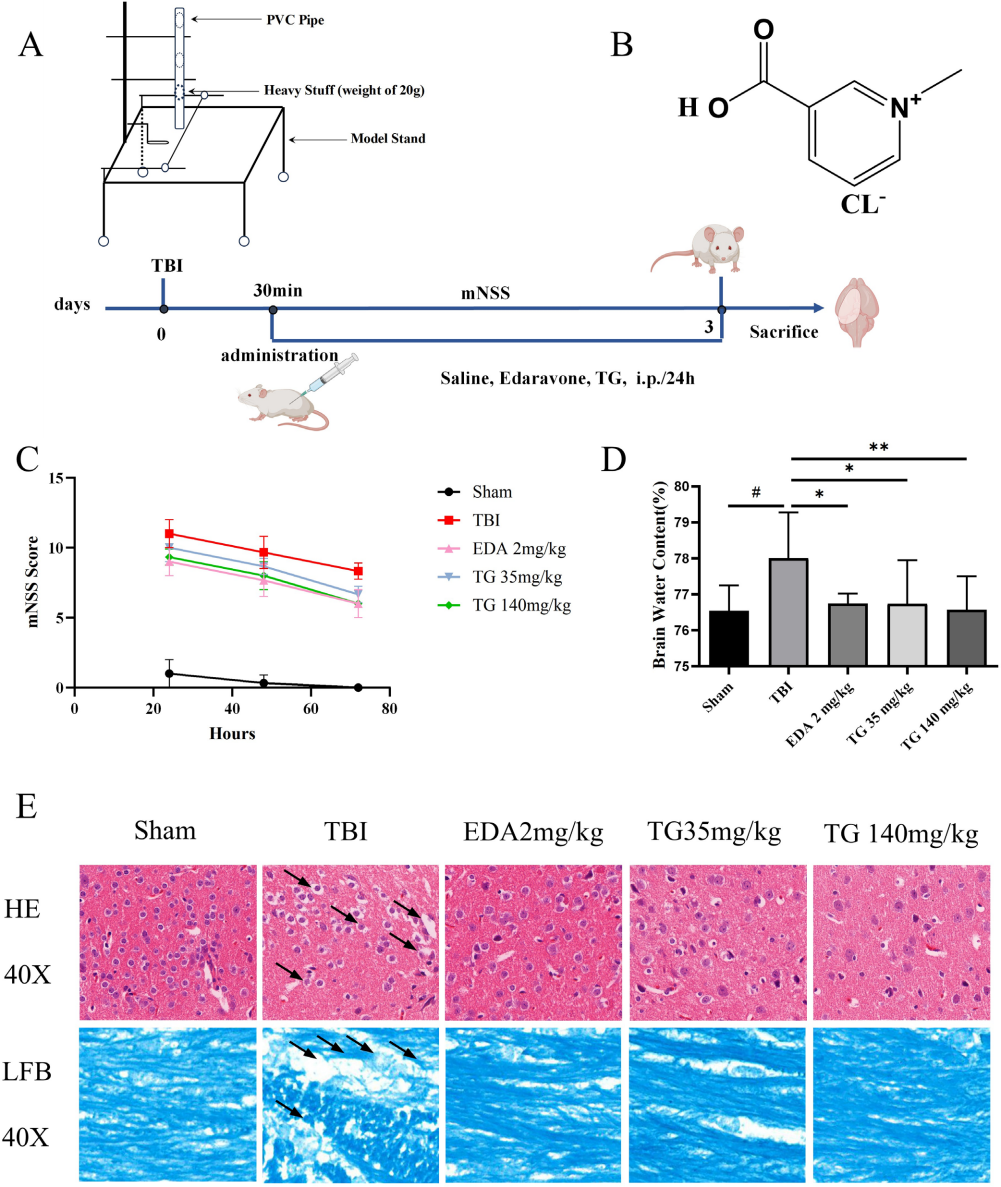
TG alleviates brain injury in TBI mice. TG was administered intraperitoneally at 35 or 140 mg/kg to mice 30 min after TBI. 3 days after TBI, brain tissue samples were harvested for two primary analyses: Quantification of edema (brain water content) and histopathological assessment (HE and LFB staining). (A) Schematic of the experimental timeline. (B) Molecular structure of TG. (C) Neurological deficit scores of the experimental groups. (D) Statistical results of brain water content measurement (*n* = 10). #*p* < 0.05 vs. sham group; *p < 0.05, *p* < 0.01 vs. TBI group. (E) Representative micrographs illustrating histological features via HE and LFB staining (original magnification, 40×).

To systematically evaluate the effects of TG on neurological function, the mNSS was employed. Compared with the TBI group, TG‐treated mice exhibited significantly lower mNSS scores during the acute post‐injury period, indicating attenuation of neurological deficits (Figure 4C).

Brain edema was evaluated by measuring brain water content. TG administration notably reduced brain water content in comparison to the TBI group, indicating its efficacy in alleviating cerebral edema during the acute phase following injury (Figure 4D). Histopathological analysis via HE staining revealed obvious edema, variable‐sized vacuoles, and neuronal pyknosis in the TBI group (Figure 4E). These pathological alterations were substantially alleviated in TG‐treated mice. LFB staining further showed that myelin staining in the corpus callosum was weakened in the TBI group, accompanied by blank areas and “vacuole‐like” changes (Figure 4E). Treatment with trigonelline notably restored the structural integrity of the myelin.

Collectively, these findings suggest that trigonelline effectively attenuates TBI‐induced secondary injury, improves cerebral histopathology, and alleviates neurological deficits during the acute phase following injury.

### 3.3 Trigonelline Attenuates Oxidative Stress and Inflammation Following TBI

**FIGURE 5:**
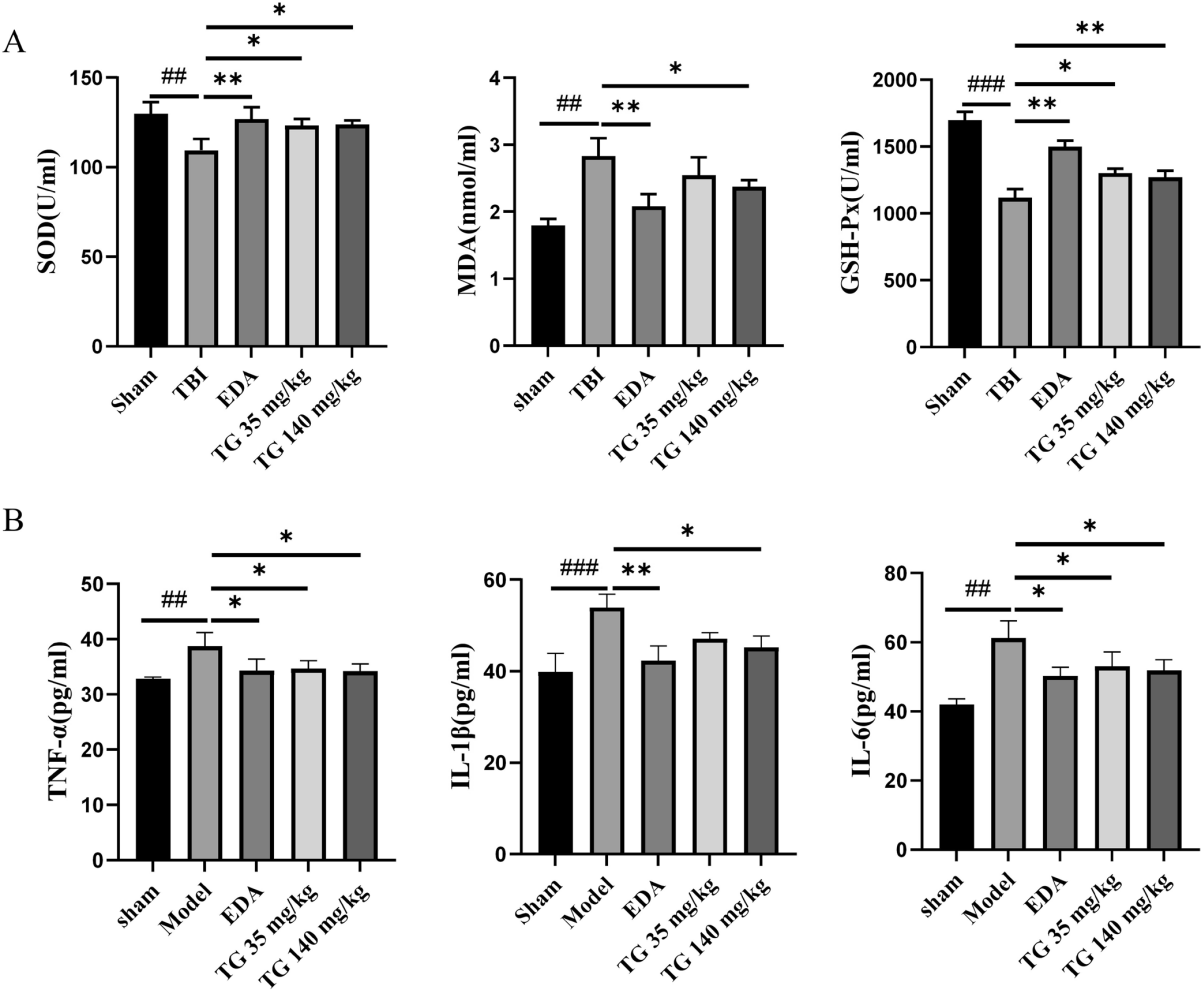
TG mitigates cerebral oxidative stress and neuroinflammation post‐TBI. (A) ELISA‐based quantification of SOD, MDA, and GSH‐Px in brain homogenates. (B) Assessment of pro‐inflammatory cytokine concentrations (TNF‐α, IL‐1β, IL‐6) in brain tissue lysates via ELISA. *p < 0.05, **p < 0.01.

Compared with the TBI group, TG administration (35 and 140 mg/kg) significantly enhanced SOD and GSH‐Px activities while reducing MDA levels (*p* < 0.05 or *p* < 0.01) (Figure 5A).

Furthermore, ELISA analysis showed that TG significantly attenuated TNF‐α, IL‐1β, and IL‐6 expression in TBI mice (*p* < 0.05, *p* < 0.01) (Figure 5B). These findings indicate that TG attenuates oxidative damage and inflammatory cascades during the acute phase following TBI.

### 3.4 Trigonelline Modulates MMP9 and AQP4 Expression in Injured Brain Tissue

**FIGURE 6:**
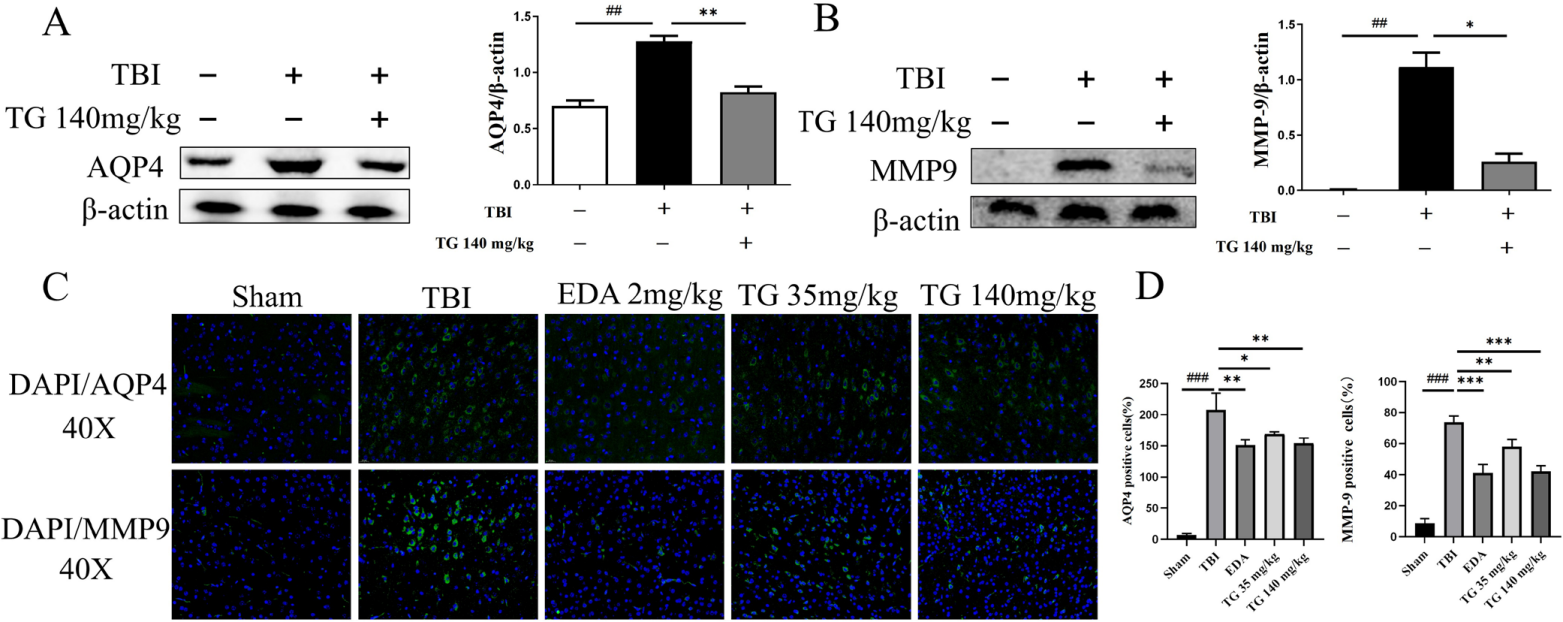
Effect of TG on AQP4 and MMP9 protein expression. (A) Confocal laser scanning microscopy images showing the distribution and expression of AQP4 and MMP9 (green fluorescence) and nuclei (DAPI, blue) in mouse brain tissue. (B) Quantitative analysis of fluorescence intensity for AQP4 and MMP9 across different experimental groups. (C) WB analysis of AQP4 protein in mouse brain tissue with quantitative densitometry. (D) Quantification of MMP9 protein expression from Western blot assays. ^##^ *p* < 0.01, ^###^ *p* < 0.001 vs. control; *p < 0.05, **p < 0.01 vs. TBI.

Western blot results demonstrated that MMP9 and AQP4 expression was significantly increased following TBI (Figure 6A,B), whereas TG treatment markedly suppressed their expression.

Immunofluorescence staining further confirmed the findings, showing parallel changes in MMP9 and AQP4 localization and fluorescence intensity (Figure 6C,D).

Overall, these data indicate that TG may alleviate acute cerebral edema and BBB‐related pathological alterations by suppressing MMP9 and AQP4 expression.

### 3.5 Trigonelline Attenuates Neuronal Apoptosis Induced by TBI

**FIGURE 7:**
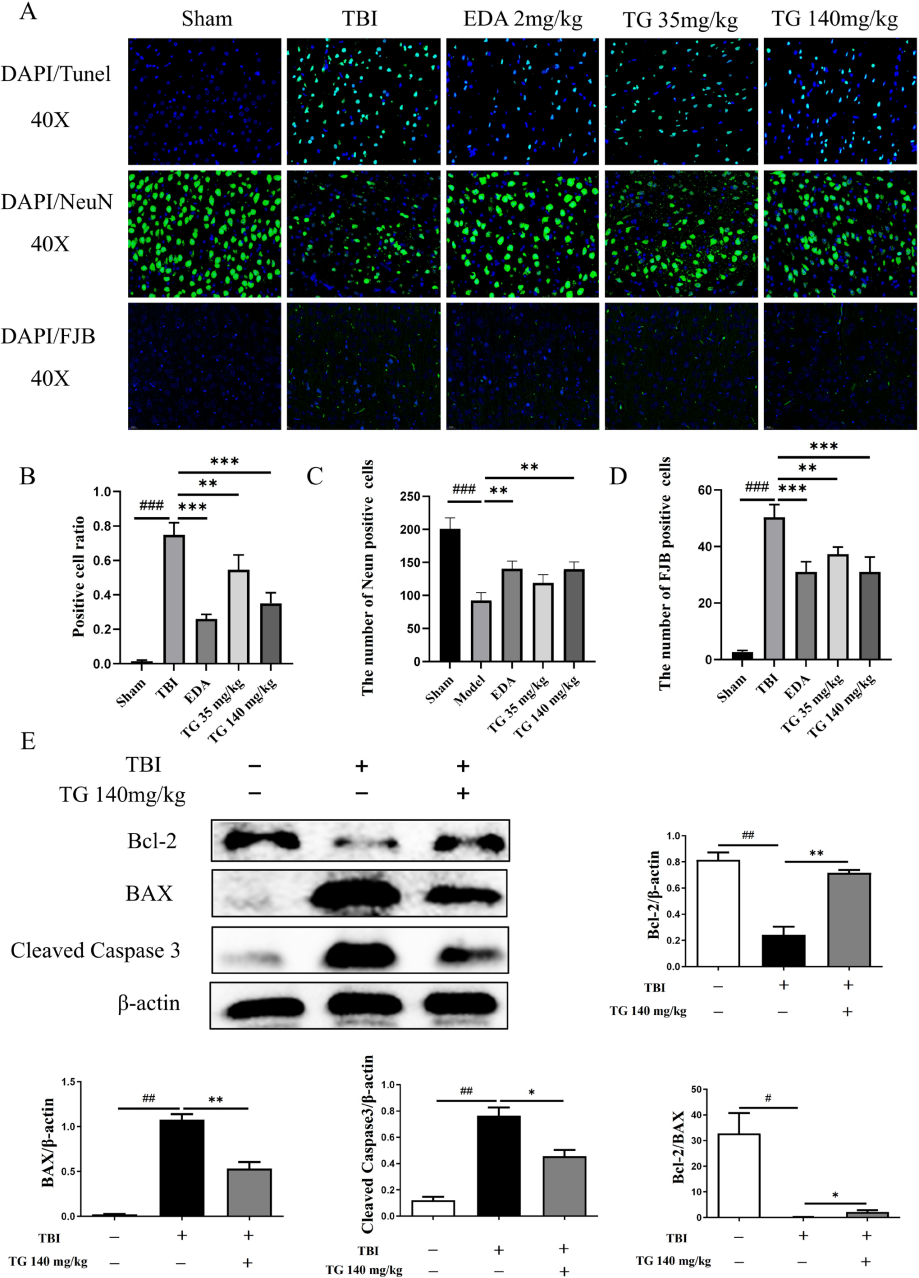
TG Attenuates Neuronal Apoptosis in TBI Brain Tissue. (A) Representative images of TUNEL, NeuN, and FJB staining in mouse brain tissue. Green fluorescence indicates TUNEL‐positive cells, NeuN, and FJB signals; blue represents DAPI‐stained nuclei (400×). (B) Measurement of TUNEL‐positive neurons. (C) Assessment of NeuN‐positive neuronal counts. (D) Quantification of FJB‐positive neurons. (E) WB and quantification of Bcl‐2, Bax, and Cleaved Caspase‐3 in brain tissues. Data are mean ± SD. #*p* < 0.05, ##*p* < 0.01, ###*p* < 0.001 vs. sham; *p < 0.05, **p < 0.01, ***p < 0.001 vs. TBI.

Neuronal apoptosis was assessed via TUNEL/NeuN/FJB staining. The therapy significantly reduced the number of TUNEL‐positive neurons in the injured cortex compared with the TBI group (Figure 7A,B). NeuN staining demonstrated a marked loss of neurons following TBI (Figure 7A,C), whereas TG intervention significantly increased NeuN expression. Consistently, FJB staining revealed extensive neuronal degeneration in the TBI group, which was significantly attenuated by TG administration.

Western blot analysis showed that TBI significantly decreased BCL‐2 expression while increasing Bax and cleaved caspase‐3 levels. TG treatment reversed these changes and significantly reduced the Bax/BCL‐2 ratio (Figure 7E), indicating suppression of apoptosis during the acute post‐injury period.

### 3.6 Trigonelline Exerts Neuroprotective Effects via the MAPK Pathway

PPI network analysis identified ERK, p38, and JNK as key regulatory nodes. GO and KEGG analyses showed that TG primarily modulates inflammatory responses and is closely associated with the MAPK signaling pathway.

**FIGURE 8:**
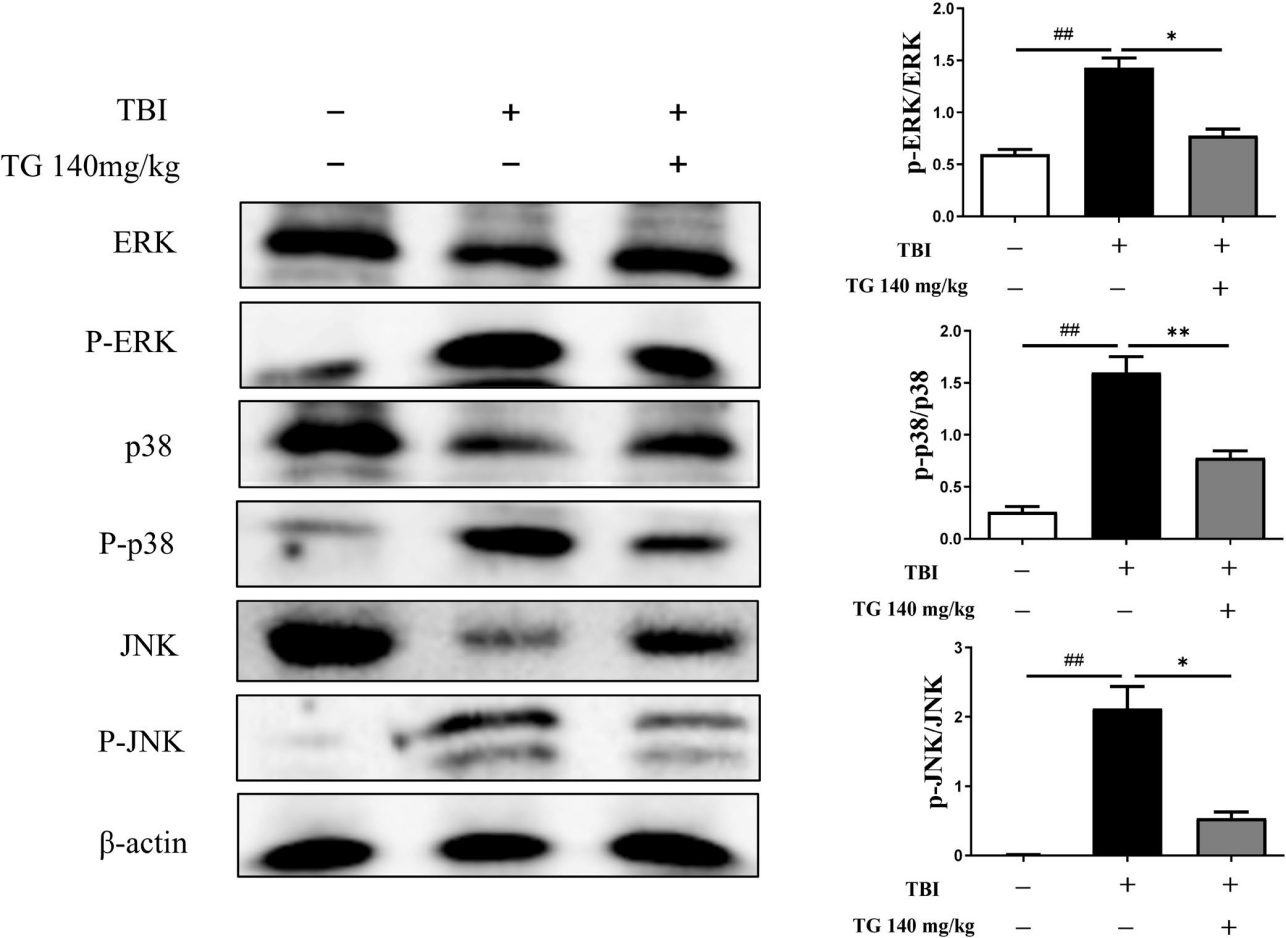
Effect of TG on ERK/p38/JNK‐related protein expression. Western blot profiles of total/phosphorylated ERK, p38, and JNK in the TBI mouse brain. Bar graphs depict mean ± SD. Statistical significance: *p < 0.05, **p < 0.01 vs. sham; ##*p* < 0.01 vs. TBI.

Consistent with these predictions, Western blot analysis demonstrated that TBI markedly increased the phosphorylation levels of ERK, p38, and JNK, whereas TG treatment significantly attenuated their hyperphosphorylation (Figure 8). Considering the parallel downregulation of AQP4 and MMP9, these results suggest that TG may exert anti‐inflammatory and neuroprotective effects during the acute phase of TBI through modulation of MAPK signaling and related downstream targets.

## 4 Discussion

Secondary injury following TBI involves complex interrelated mechanisms, including oxidative stress, inflammatory responses, apoptosis, cerebral edema, and disruption of the BBB, which collectively contribute to acute neurological dysfunction [20, 21]. Currently, no universally effective pharmacological interventions for TBI exist, highlighting the need for novel therapeutics with well‐characterized mechanisms of action. This study aimed to evaluate the neuroprotective effects of TG during the acute phase of TBI and uncover the mechanisms involved.

Accumulating evidence indicates that TG modulates key inflammatory and oxidative stress‐related pathways implicated in neuronal injury [22]. Experimental studies have reported that TG suppresses pro‐inflammatory cytokine production, reduces lipid peroxidation, and enhances endogenous antioxidant capacity, thereby conferring neuroprotection in models of neuroinflammation and cognitive dysfunction [23, 24]. In the present study, TG treatment within 72 h after injury ameliorated histopathological damage, reduced cerebral edema, and improved acute neurological deficits in a weight‐drop TBI mouse model. Network pharmacology and molecular docking identified IL6, ALB, TNF, IL1B, MMP9, and AQP4 as potential targets, implicating MAPK, PI3K‐Akt, and TNF pathways in TG‐mediated effects.

Oxidative stress and neuroinflammation are central contributors to secondary brain injury following TBI [25, 26]. Excessive ROS promote lipid peroxidation, membrane disruption, and neuronal apoptosis, while elevated TNF‐α, IL‐1β, and IL‐6 levels exacerbate neuronal damage [27, 28, 29]. Increasing evidence indicates that TG exerts pronounced antioxidant and anti‐inflammatory effects in experimental models of neurological and systemic inflammatory disorders [30, 31], including attenuation of lipopolysaccharide‐induced cognitive impairments via regulation of oxidative stress, inflammation, and AChE activity [32]. In line with these observations, our results demonstrate that TG significantly decreased MDA levels and increased SOD and GSH‐Px activities, indicating attenuation of oxidative stress. Concurrently, TG markedly suppressed TNF‐α, IL‐1β, and IL‐6 expression, supporting its anti‐inflammatory actions during the acute phase of TBI.

Apoptosis is a key contributor to secondary neuronal loss following TBI, and excessive activation of apoptotic pathways exacerbates neural injury [33]. The mitochondrial apoptotic pathway, characterized by cytochrome c release and caspase‐9/3 activation, plays a pivotal role in this process [34]. ERK, JNK, and p38, components of the MAPK signaling cascade, are central regulators of both inflammatory processes and apoptotic pathways [35, 36]. Aberrant MAPK activation promotes neuronal apoptosis through upregulation of caspase‐3 and downregulation of anti‐apoptotic proteins such as BCL‐2 [37, 38], whereas MAPK inhibition has been shown to attenuate neuroinflammation and improve functional recovery in TBI models [39, 40]. In accordance with network pharmacology predictions, our results demonstrate that TG effectively attenuated ERK, JNK, and p38 activation, accompanied by increased BCL‐2 and decreased BAX and Cleaved Caspase‐3 levels. These findings suggest that TG exerts acute neuroprotection by modulating MAPK‐mediated inflammatory and apoptotic signaling.

**FIGURE 9:**
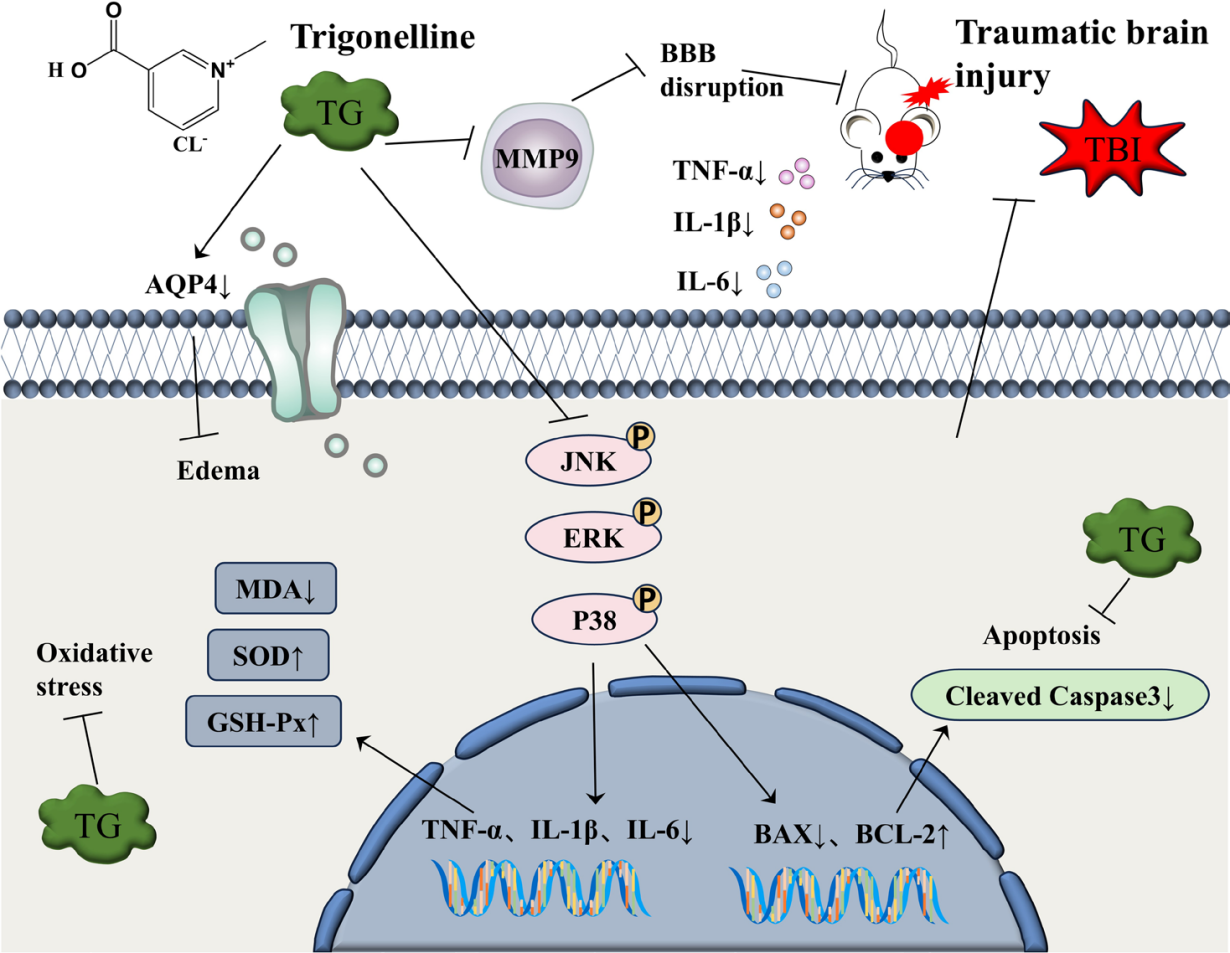
Diagram depicting trigonelline's mechanism of action in TBI treatment.

Cerebral edema and BBB disruption are major pathological features of acute TBI and are strongly associated with poor clinical outcomes [41, 42]. AQP4, a major CNS water channel protein enriched in astrocytic end‐feet, plays a critical role in cytotoxic edema by regulating water homeostasis in the injured brain [43, 44, 45]. In the present study, TG treatment downregulated AQP4 expression, suggesting a potential contribution to the attenuation of cytotoxic edema during the acute post‐injury period. BBB disruption further exacerbates secondary injury by facilitating immune cell infiltration and amplifying neuroinflammation [46, 47, 48]. Oxidative stress–induced activation of matrix metalloproteinases, particularly MMP‐9, contributes to BBB breakdown through degradation of extracellular matrix components [49, 50]. This study shows that MMP‐9 expression was significantly elevated at 72 h post‐TBI and was effectively suppressed by TG, indicating partial preservation of BBB structure and function (Figure 9). From a translational perspective, the potential clinical applicability of TG warrants careful consideration. Preclinical studies suggest that TG exhibits low acute toxicity, with a high LD_50_ in rodents and no apparent organ toxicity following repeated administration at moderate doses [51]. However, systematic evaluations of chronic toxicity, reproductive safety, and context‐dependent adverse effects remain limited [52]. Pharmacokinetic studies indicate that TG is orally absorbable and systemically available in animal models, yet its metabolic profile, blood–brain barrier permeability, and human pharmacokinetics have not been fully characterized [53]. These factors underscore the necessity for comprehensive pharmacokinetic and safety assessments prior to clinical translation.

Despite these promising findings, several limitations exist. First, this study focused on the acute phase (within 72 h post‐TBI), and the long‐term efficacy of TG remains unknown. Second, although MAPK signaling, AQP4, and MMP‐9 were highlighted as key mediators, other pathways may contribute to TG's effects and warrant further investigation. Finally, while the mouse model of TBI provides mechanistic insights, it does not fully recapitulate the complexity of human TBI, emphasizing the need for validation in higher‐order animal models and, ultimately, clinical studies.

## 5 Conclusion

In summary, TG exhibits significant neuroprotective effects in a mouse model of TBI during the acute phase. These effects are associated with attenuation of oxidative stress and inflammatory responses, inhibition of MAPK‐mediated apoptotic signaling, and downregulation of AQP4 and MMP‐9, leading to reduced cerebral edema and partial preservation of BBB integrity. Further studies are required to determine whether these protective effects persist into the subacute and chronic phases of TBI, and to clarify the toxicological profile, optimal dosing, and pharmacokinetics for potential clinical application.

## Author Contributions

Qian Zhang and Yuefan Zhang were responsible for the conceptualization and experimental design. Qian Zhang and Zhibing Song performed the in vivo studies and drafted the manuscript, while Zixiang Tang, Guoqiang Wang, and Xiang Yang assisted with data acquisition. The manuscript was critically reviewed and revised by Jincai Li, Tiejun Li, and Qian Zhang All authors critically reviewed the manuscript. The final version was approved by all contributors prior to submission.

## Funding

This work was supported by National Natural Science Foundation of China, 82204929. Key Specialty Construction Project of Pudong Health and Family Planning Commission of Shanghai, PWZzk2022‐08. University Synergy Innovation Program of Anhui Province, GXXT‐2023‐073. Bozhou Municipal Key Laboratory of Modern Traditional Chinese Medicine Manufacturing, 002006.

## Ethics Statement

This study was reviewed and approved by the Ethics Committee of School of Medicine, Shanghai University, China (Approval ID: ECSHU‐2024‐005), and all experimental procedures were conducted in accordance with relevant institutional guidelines and regulations.

## Conflicts of Interest

The authors declare no conflicts of interest.

## Data Availability

The data that support the findings of this study are available on request from the corresponding author. The data are not publicly available due to privacy or ethical restrictions.
